# The role of the serum 25-OH vitamin D level on detecting prostate cancer in men with elevated prostate-specific antigen levels

**DOI:** 10.1038/s41598-022-17563-8

**Published:** 2022-08-18

**Authors:** Min Hyuk Kim, Sangjun Yoo, Min Soo Choo, Min Chul Cho, Hwancheol Son, Hyeon Jeong

**Affiliations:** 1 Department of Urology, Seoul National University Boramae Medical Center, Sindaebang 2(i)-dong, Dongjak-gu, Seoul, 07061 Republic of Korea; 2 Department of Urology, College of Medicine, Seoul National University, Seoul, Republic of Korea

**Keywords:** Oncology, Urology

## Abstract

We aimed to determine whether vitamin D levels before prostate biopsy have diagnostic value for clinically significant prostate cancer. The study cohort included patients who underwent prostate biopsy. A total of 224 patients were enrolled in our study and serum vitamin D levels were measured from February 2016 to December 2019 in routine laboratory tests. To determine the relationship between vitamin D levels and aggressiveness of prostate cancer, we used logistic multivariate analysis. Based on the histopathological results of patients who underwent radical prostatectomy, the serum vitamin D level was significantly lower with the large tumor volume group. In the univariate analysis, the prostate cancer diagnosis rate was associated with low vitamin D levels. Low vitamin D level is negatively correlated with clinically significant prostate cancer (biopsy Gleason score of 7 or higher) in the univariate (Odds ratio [OR], 0.955; P < 0.001) and multivariate (OR, 0.944; P = 0.027) analyses. In conclusion, we found that the incidence of clinically significant prostate cancer might related to low vitamin D level in the Asian population. In the future, a larger population and prospective study are needed.

## Introduction

Prostate cancer is the second most common cancer diagnosed in men worldwide, with more than 1 million cases occurring each year^1^. In the United States, prostate cancer is the most common cancer in men, accounting for 26% of cancer diagnoses^2^. In addition, the incidence and prevalence of prostate cancer have recently increased in Asian countries^3^. In Korea, the incidence and prevalence of prostate cancer became fourth and third, respectively, in 2018. With the discovery of prostate-specific antigen (PSA) in the 1970s, the diagnosis rate has increased through screening for prostate cancer and early detection of prostate cancer was made possible by discovery of PSA. However, the United State Preventive Services Task Force (USPSTF) in 2012 evaluated PSA based screening for prostate cancer to grade D (The USPSTF recommends against the service. There is moderate or high certainty that the service has no net benefit or that the harms outweigh the benefits), because of concerns about overdiagnosis^4^. In addition, PSA exam did not have a clear cut off value, and when cut off value was set to 2.5 and 4.0 ng/ml, 80% of positive PSA test results were false-positive^5^. For this reason, screening for prostate cancer only with the PSA exam may perform unnecessary prostate biopsies and cause complications such as hematuria and prostatitis. Therefore, additional diagnostic tests that can screen prostate cancer together with PSA are needed.

In addition, the current clinical guidelines suggest active surveillance (AS) as a preferred treatment option for clinically insignificant prostate cancer which is low-risk prostate cancer^6,7^. Accurately detecting clinically significant prostate cancer patients who need active treatment at appropriate time, became more important than ever before. Multiparametric magnetic resonance imaging (MRI) for prostate cancer and Prostate Imaging-Reporting and Data System (PI-RADS) has greatly contributed to the differentiation of clinically significant prostate cancer before prostate biopsy. MRI fusion biopsy, which directly puncture the prostate target lesion indicated on MRI, has the effect of increasing the clinically significant prostate cancer diagnosis rate. Although multiparametric magnetic resonance imaging (MRI) and MRI fusion biopsy have gained attention for detecting clinically significant prostate cancer, due to the high cost and accessibility issues, there is a need for more cost-effective and easy methods. To this end, several studies have been conducted for appropriate predictive value using PSA level such as PSA density, PSA density of transition zone, free/total PSA ratio, and PSA velocity, but diagnostic parameters with high sensitivity and specificity have not yet been determined^8^.

Recent studies have shown that vitamin D deficiency is linked to several types of cancer (e.g. breast, colon, rectum, stomach, and esophageal cancers)^9–12^. Several studies have shown that vitamin D levels and cancer incidence are negatively correlated and that low vitamin D levels are related to overall survival and disease-free survival. These anticancer effects of vitamin D are mediated by the regulation of specific signaling pathways via vitamin D receptors (VDRs). The activated form of vitamin D, calcitriol, binds to VDR to promote cell differentiation and apoptosis and inhibit cellular proliferation, angiogenesis, and tumor cell invasion^13–16^. In addition, vitamin D also affects the immune system, causing anti-inflammatory reactions and suppressing cancer progression^17–19^.

Several studies have revealed a negative relationship between vitamin D and prostate cancer. Moreover, a previous study revealed a negative relationship between vitamin D and Gleason score (GS), which would be useful for prostate cancer aggressiveness evaluation^20,21^. Although the association between prostate cancer and vitamin D has been reported, few studies have investigated the diagnostic value of the vitamin D levels in clinically significant prostate cancer. Therefore, in the current study, we aimed to reveal the role of serum vitamin D, the precursor for calcitriol, in the detection of prostate cancer, especially in the detection of clinically significant prostate cancer. In addition, there was a study showing that MRI lesions with multiple sclerosis patients had a significantly correlated inversely with serum vitamin levels^22^. Since vitamin D may show different imaging findings on MRI, we analyzed the vitamin D level and the presence of suspected prostate cancer target lesion to confirm whether vitamin D can help diagnose prostate cancer even in patients who have undergone multiparametric MRI.

## Materials and methods

We established a study cohort of patients who underwent transrectal ultrasound (TRUS)-guided prostate biopsy after outpatient treatment at the Department of Urology at Boramae Medical Center, Seoul, Republic of Korea. These patients consistently had a PSA level of ≥ 3 ng/mL, had suspected cancer lesions on imaging tests, or had nodules on digital rectal examination. From December 2015 to December 2019, 1500 patients underwent TRUS-guided prostate biopsy, among whom patients previously treated for prostate cancer, and patients suspected of regional lymph node metastasis or distant metastasis on the imaging test were excluded. Since prostate cancer patients with suspected metastatic lesions have very high PSA levels, which may underestimate the effects of factors other than PSA. Finally, 224 individuals who allowed their serum vitamin D levels to be measured before TRUS-guided prostate biopsy were included in our study. This study was approved by the institutional review board of our hospital.

Prostate cancer with a biopsy Gleason score (GS) of ≥ 7 was defined as clinically significant prostate cancer. In these patients, standard 12-core TRUS-guided prostate biopsy was routinely performed, although for areas where prostate cancer was strongly suspected on imaging studies, one to two biopsies were additionally performed. In these patients, serum vitamin D levels were routinely measured 1–4 weeks before TRUS-guided prostate biopsy. In our center, we assessed serum 25-OH vitamin D levels using a chemiluminescent protein binding assay. Because there was no seasonal variation in vitamin D in our study patients, we decided to conduct the analysis regardless of the season (Supplement 1). For the logistic regression analysis, the vitamin D level was used as a continuous variable.

To compare the serum vitamin D levels according to pathohistological results of TRUS-guided prostate biopsy, the ratio of the total number of cores to the number of positive cores was calculated and divided into high and low groups with an average value of 43.4%. The serum vitamin D levels were compared in 36 patients who underwent radical prostatectomy (RP) by the final pathology result (tumor volume, lymphatic invasion, and perineural invasion factor). The final pathology result was based on pathologic T stage 3 (pT3), and the tumor volume ratio was divided into large and small groups based on an average value of 17.2%. Since 36 patients were small cohort, we analyzed it after confirming the normality test and equivariance test.

Prostate MRI was performed with Siemens VIDA 3.0 T multiparametric MRI for high resolution. Multiparametric MRI discriminates prostate cancer from normal prostate tissue by combining anatomical information from T1- and T2-weighted sequences and functional information from diffusion-weighted imaging (DWI) and dynamic contrast enhancement (DCE). The sequence is as follows. T2 TSE (Turbo spin echo) sagittal view, axial view, T1 TSE oblique axial view, and T2 TSE coronal view are obtained. After that, the oblique axial view of diffusion weighted image (DWI) (b-value 0, 50, 1000, 1400 s/mm^2^) was acquired, and the apparent diffusion coefficient (ADC) image was obtained by dividing it into b-values 50,1000 s/mm^2^ and 50,1000, 1400 s/mm^2^. In order to obtain T1 TWIST VIBE Dynamic oblique axial (20 Dynamic) image, the contrast media (gadoterate meglumine) is injected (0.1 cc/kg) when moving from dynamic phase 1 to 2 and a flow rate of 2 ml/s is injected with an auto injector. T1 TSE fat suppression axial view is obtained after contrast enhancement^23^. Multiparametric MRI was performed in all patients with prostate cancer after the histological diagnosis of prostate cancer, and suspicious lesions on MRI were checked by a urology-specialized radiologist if there was a lesion with a PI-RADS version 2 score of ≥ 3.

The distribution of patient characteristics was compared using Student’s t-test, Pearson’s χ^2^ test, or Fisher’s exact test. We present the demographic and clinical characteristics of patients by detecting prostate cancer. Continuous factors are expressed as means and standard deviations, and categorical factors are expressed as percentages. In addition, the detection rate of prostate cancer and clinically significant prostate cancer according to the vitamin D levels was presented and compared. We performed logistic regression models for univariate and multivariate analyses to determine the relationship between prostate cancer and clinically significant prostate cancer and vitamin D level, in addition to other variables, including age, body mass index (BMI), hypertension (HTN), diabetes, serum PSA level, and prostate volume. All statistical comparisons were performed using the IBM SPSS Statistics version 21. Statistical significance was set at P < 0.05.

### Ethical approval

The study was approved by the Ethics Committee of the Seoul National University Boramae Medical Center (10-2021-123).

### Consent to participate and for publication

Data were collected retrospectively from clinical files and anonymized for analysis. No specific consent was obtained from all patients.

## Results

Age (non-cancer group 66.5 years vs. clinically insignificant prostate cancer [insignificant] 69.3 years vs. clinically significant prostate cancer [significant] 69.2 years, P = 0.049) and PSA (non-cancer group 7.9 ng/mL vs. insignificant 9.7 ng/mL vs. significant 34.2 ng/mL, P < 0.001) were higher in the prostate cancer group, and the prostate total volume (non-cancer group 48.9 mL vs. insignificant 34.2 mL vs. significant 35.0 mL, P < 0.001) was lower in men with prostate cancer than in men without prostate cancer. In particular, the clinically significant prostate cancer group had significantly higher PSA levels than the non-cancer group and clinically insignificant prostate cancer group. A Hypertension factor (non-cancer group 40.1% vs. insignificant 55.0% vs. significant 55.7%, P = 0.085) and diabetes factor (non-cancer group 13.4% vs. insignificant 30.0% vs. significant 24.6%, P = 0.055) also showed marginal differences between the three groups. The serum vitamin D levels were lower in the prostate cancer group, but the difference was not statistically significant (non-cancer group 19.6 ng/mL vs. insignificant 19.1 ng/mL vs. significant 18.1 ng/mL, P = 0.562) (Table 1).

**Table 1:** Demographic and clinical characteristics of patients.

	Non-cancer group (*n* = 142)	Insignificant (*n* = 20)	Significant (*n* = 62)	*p* value
Age (years)	66.5 ± 7.9	69.3 ± 7.2	69.2 ± 8.7	0.049
BMI (kg/m^2^)	24.8 ± 2.9	23.9 ± 2.3	24.4 ± 3.3	0.383
Hypertension (n, %)	57 (40.1)	11 (55.0)	34 (55.7)	0.085
Diabetes (n, %)	19 (13.4)	6 (30.0)	15 (24.6)	0.055
WBC (× ${10}^{3}/\mathrm{\mu L}$)	6.1 ± 2.6	5.5 ± 1.2	6.1 ± 1.5	0.561
ESR (mm/h)	11.9 ± 11.2	6.2 ± 7.2	14.9 ± 15.2	0.328
CRP (mg/L)	0.36 ± 1.08	0.11 ± 0.14	0.28 ± 0.65	0.679
PSA (ng/mL)	7.9 ± 5.7	9.7 ± 8.3	34.2 ± 66.7	< 0.001
Prostate total volume (g)	48.9 ± 22.8	34.2 ± 18.8	35.0 ± 16.1	< 0.001
Serum vitamin D level (ng/mL)	19.6 ± 10.0	19.1 ± 7.5	18.1 ± 8.5	0.562

*BMI* body mass index, *WBC* white blood cell, *ESR* erythrocyte sedimentation rate, *CRP* c-reactive protein, *PSA* prostate-specific antigen.

Of the 82 patients with prostate cancer, 36 patients underwent RP, and the remaining patients underwent radiotherapy or androgen deprivation therapy. On Table 2, we compared serum vitamin D levels according to histopathology in 224 patients who underwent TRUS-guided prostate biopsy and 36 patients who underwent radical prostatectomy (RP). In TRUS-guided prostate biopsy, the ratio of the total number of cores to the number of positive cores was calculated. The serum vitamin D levels were marginally lower in the group with a high positive core ratio than in the group with a low positive core ratio (high group 16.3 ng/mL vs. low group 19.5 ng/mL, P = 0.087). According to the histopathological results after RP, the serum vitamin D level was marginally lower in patients with pT3 or higher than in patients with pT2 or lower (pT2 group ≥ 19.1 ng/mL vs. pT3 group ≤ 15.0 ng/mL, P = 0.089). The serum vitamin D level was low in the group with a large volume ratio of prostate cancer (large group 11.2 ng/mL vs. small group 19.2 ng/mL, P < 0.001). In the presence of lymphatic and perineural invasions, the serum vitamin D levels were lower in the invasion group, but the difference was not statistically significant (lymphatic invasion (−)group 18.2 ng/mL vs. lymphatic invasion (+)group 14.8 ng/mL, P = 0.278; perineural invasion (−)group 19.3 ng/mL vs. perineural invasion (+) group 16.7 ng/mL, P = 0.300) (Table 2).

**Table 2:** Serum vitamin D level according to pathohistological results.

	Serum vitamin D level (ng/mL)	*p* value
**Patients with TRUS-guided prostate biopsy (*n* = 82)**		
Positive/total core (%)		0.087
Low (*n* = 51)	19.5 ± 7.4	
High (*n* = 31)	16.3 ± 9.2	
**Patients with Radical prostatectomy (*n* = 36)**		
Pathology		0.089
pT2 ≥ (*n* = 23)	19.1 ± 7.1	
pT3 ≤ (*n* = 13)	15.0 ± 6.0	
Tumor volume (%)		< 0.001
Small (*n* = 29)	19.2 ± 6.8	
Large (*n* = 7)	11.2 ± 3.2	
Lymphatic invasion		0.278
No (*n* = 30)	18.2 ± 7.2	
Yes (*n* = 6)	14.8 ± 5.2	
Perineural invasion		0.300
No (*n* = 13)	19.3 ± 8.1	
Yes (*n* = 23)	16.7 ± 6.2	

*pT2* pathologic T2 stage, *pT3* pathologic T3 stage.

In the univariate analysis, the prostate cancer diagnosis rate was significantly increased with low vitamin D levels (Odds ratio [OR], 0.975; P < 0.001) and other factors such as small prostate total volume (OR, 0.983; P < 0.001), young age (OR, 0.993; P < 0.001), low BMI (OR, 0.978; P < 0.001), and high serum PSA levels (OR, 1.063; P < 0.001). In the multivariate analysis, the prostate cancer diagnosis rate was significantly increased with high serum PSA levels (OR, 1.083; P = 0.003) and small prostate total volume (OR, 0.937; P < 0.001). The serum vitamin D levels were not statistically significant but were marginally associated with the prostate cancer diagnosis rate (OR, 0.963; P = 0.094) (Table 3A).

**Table 3:** Univariate and multivariate logistic regression analysis of Asian patients with prostate biopsy (*n* = 171).

	Univariate (OR 95% CI)	*p* value	Multivariate (OR 95% CI)	*p* value
**(A) Logistic regression analysis according to biopsy Gleason score 6**				
Age (continuous variable)	0.993 (0.989–0.997)	< 0.001	1.064 (1.012–1.118)	0.015
BMI (continuous variable)	0.978 (0.968–0.989)	< 0.001		
HTN (yes vs no)	0.821 (0.556–1.213)	0.323		
DM (yes vs no)	1.105 (0.594–2.056)	0.752		
PSA (continuous variable)	1.063 (1.027–1.100)	< 0.001	1.083 (1.028–1.140)	0.003
Prostate total volume (continuous variable)	0.983 (0.976–0.990)	< 0.001	0.937 (0.910–0.965)	< 0.001
Serum Vitamin D level (continuous variable)	0.975 (0.962–0.988)	< 0.001	0.963 (0.922–1.006)	0.094
**(B) Logistic regression analysis according to biopsy Gleason score 7 (clinically significant prostate cancer)**				
Age (continuous variable)	0.987 (0.982–0.991)	< 0.001	1.026 (1.002–1.050)	0.032
BMI (continuous variable)	0.962 (0.950–0.973)	< 0.001		
HTN (yes vs no)	0.500 (0.331–0.755)	0.001		
DM (yes vs no)	0.600 (0.316–1.138)	0.118		
PSA (continuous variable)	1.064 (1.031–1.099)	< 0.001	1.077 (1.031–1.125)	0.001
Prostate total volume (continuous variable)	0.974 (0.965–0.982)	< 0.001	0.933 (0.902–0.966)	< 0.001
Serum Vitamin D level (continuous variable)	0.955 (0.941–0.969)	< 0.001	0.944 (0.897–0.993)	0.027

*BMI* body mass index, *HTN* hypertension, *DM* diabetes mellitus, *PSA* prostate-specific antigen, *CI* confidence interval.

We also analyzed the correlation between the vitamin D level and clinically significant prostate cancer diagnosis. In the univariate analysis, the clinically significant prostate cancer diagnosis rate was significantly increased with low vitamin D levels (OR, 0.955; P < 0.001) and other factors such as small prostate total volume (OR, 0.974; P < 0.001), young age (OR, 0.987; P < 0.001), low BMI (OR, 0.962; P < 0.001), and no HTN (OR, 0.500; P = 0.001). In the multivariate analysis, the clinically significant prostate cancer diagnosis rate was significantly increased with low vitamin D levels (OR, 0.944; P = 0.027) and other factors such as old age (OR, 1.026; P = 0.032), high PSA levels (OR, 1.077; P = 0.001), and small prostate total volume (OR, 0.933; P < 0.001) (Table 3B).

Because there was a seasonal variation in the serum vitamin D levels, patients were divided according to the season. Of the 224 patients, 101 underwent TRUS-guided prostate biopsy in the spring, 40 in the summer, 24 in the fall, and 59 in the winter. However, there was no seasonal difference in the patients in our study (P = 0.551), which may be due to increased indoor activities and increased oral intake of vitamin D (Supplement 1).

## Discussion

Currently, PSA is used as a screening test to diagnose prostate cancer, and TRUS-guided prostate biopsy is performed as a confirmation test. In a previous meta-analysis study, the sensitivity (72.1%) and positive predictive value (25.1%) of PSA for prostate cancer diagnosis were not high^24^. According to the results of recently conducted large randomized clinical trials, the proportion of clinically insignificant prostate cancer diagnosed by PSA screening was high, and as a result, several unnecessary TRUS-guided prostate biopsy were performed^25^. To overcome this, multiparametric prostate MRI is performed before TRUS-guided prostate biopsy, and MRI fusion biopsy is performed if there are target lesions, albeit with high cost and accessibility issues.

Prostate cancer is a slow-growing cancer, and asymptomatic prostate cancer is found in several individuals at autopsy. For patients with clinically insignificant prostate cancer, active surveillance is implemented to minimize the side effects of treatment without affecting their survival. With an increase in the number of patients undergoing active surveillance, it is becoming increasingly important to accurately diagnose patients with clinically significant prostate cancer who require treatment at an appropriate time. In the current study, we revealed that the serum vitamin D may be a helpful indicator for improving the detection rate of clinically significant prostate cancer. In other words, although further larger studies are needed for confirmation, serum vitamin D may be a useful way to reduce unnecessary TRUS-guided prostate biopsy and increase the diagnosis rate of clinically significant prostate cancer.

Based on our study findings, there was a significant negative correlation between vitamin D and clinically significant prostate cancer (OR, 0.944; P = 0.027). Likewise, other studies have shown a negative relationship between vitamin D and prostate cancer malignancy. In a nested case–control study, Schenk et al.^21^ recruited and analyzed 1700 cases and controls. The serum vitamin D levels were divided into quartiles and compared, and multiple regression analysis was performed by adjusting for age and race. It was found that higher the vitamin D level, lower was the risk of a GS of 8–10 (quartile 4 vs. 1; OR, 0.55; P = 0.04). In a prospective study, Ahn et al.^26^ compared 749 patients with prostate cancer with 781 control individuals and concluded that a lower vitamin D level did not increase the prostate cancer risk but could increase aggressiveness of prostate cancer (GS sum of ≥ 7 or clinical stage III or IV). Nyame et al.^27^ analyzed the correlation between the serum vitamin D level and prostate cancer aggressiveness using multiple regression analysis in 190 patients who underwent RP. Adverse pathology was defined as dominant Gleason pattern 4, the presence of any pattern 5, and extracapsular extension based on histopathology after surgery. Multiple regression analysis showed that the risk of adverse pathology was increased in the group with a serum vitamin D level of ≤ 30 ng/mL (OR, 2.64; P = 0.01).

In addition to in vitro studies, in vivo studies have shown the relationship between vitamin D and prostate cancer in human or animal cells. Oades et al.^28^ showed that VDR expression and vitamin D inhibited cancer cell growth in rodent cells, and Peehl et al.^29^ showed antiproliferative and a differentiating action of vitamin D in human prostate cancer cells. The anticancer effect of vitamin D is unclear, but animal studies have shown that it inhibits angiogenesis^30,31^. Based on these results, vitamin D can be assumed to inhibit tumor cell proliferation by inhibiting angiogenesis in prostate cancer cells. In addition, there is a theory that vitamin D binds to VDR and directly induces cell cycle arrest, thereby inhibiting tumor cell proliferation. In our study, the serum vitamin D level was higher in the group with a small tumor volume, which can be seen as a result of angiogenesis inhibition or cell cycle arrest through VDR (Table 2). The results of in vitro and in vivo studies support the results of our study, and vitamin D may be used as a tool to prevent clinically significant prostate cancer by reducing prostate malignancies. Moreover, vitamin D may be a helpful marker for detecting clinically significant prostate cancer.

We included hypertension (HTN) and diabetes mellitus (DM) factors in multivariate logistic regression analysis. Liang et al.^32^ conducted systematic review and meta-analysis by synthesizing 21 studies published until 2016, and found that the incidence of prostate cancer in individuals with HTN significantly increased (Relative risk [RR] 1.08, 95% confidence interval [CI] 1.02–1.15, P = 0.014). Kasper et al.^33^ performed meta-analysis using 19 studies published up to 2005 and found an inverse association between DM and prostate cancer, unlike HTN (RR 0.84, 95% CI 0.76–0.93, P ≤ 0.01). In our study, only the HTN factor result was significant in univariate analysis based on the biopsy Gleason score of 7, but it was not consistent with the results of previous studies (Table 3).

There have been several previous studies that BMI is related to the incidence of prostate cancer and the oncological outcome of prostate cancer^34,35^. In current study, there was an association between BMI and prostate cancer or clinically significant prostate cancer in the univariate analysis, but was excluded from the multivariate analysis results. As a result of further analysis, we found that there was an association between BMI and total prostate volume and BMI factor was excluded from the results of multivariate analysis. Wang et al.^36^ performed a meta-analysis using the results of 19 studies, and the odds ratio of benign prostate hyperplasia (BPH) was 1.21 (95% CI 1.00–1.46, P < 0.001) in the group with high BMI compared to the group with low BMI. Lee et al.^37^ recruited 146 men over 40 years of age without obesity-related metabolic disease and found that the prostate size in the BMI 25 kg/m^2^ group was larger than in the 18.5–22.9 kg/m^2^ group (18.8 ± 5.0 vs 21.8 ± 5.6, P < 0.05). There are several previous studies showing that obese patients with high BMI have large prostate total volume, therefore it can be explained that BMI was excluded from the multiple regression analysis on this study.

Vitamin D is produced from the skin by exposure to ultraviolet light or absorbed through food. Because South Korea has four seasons, there is a study showing the serum vitamin D level varies by season^38^. However, in current study, there was no difference in serum vitamin D levels according to the seasons. It can be analyzed that because prostate cancer patients are older than 60 years, the production of 7-dehydrocholesterol, a precursor of vitamin D, is reduced and the production of 25-OH-vitamin D and 1,25-OH2-vitamin D is also reduced due to deterioration of liver and renal function^39^.

To increase the diagnosis rate of clinically significant prostate cancer that requires treatment, multiparametric prostate MRI is performed before TRUS-guided prostate biopsy. For patients with high PSA levels, it is useful to obtain an accurate target lesion through multiparametric prostate MRI and perform MRI fusion biopsy. However, for patients with slightly elevated PSA levels, multiparametric MRI scans that are not covered by health insurance can be a financial burden to the patient, and there are cases where it is difficult to distinguish between prostate cancer and normal prostate tissue even though standardized interpretation systems, such as PI-RADS version 2, have been adapted. Based on this study, the higher the vitamin D level, the more target lesions were observed on multiparametric prostate MRI (Supplement 2). In other words, the radiology specialists could observe the target lesion more clearly by distinguishing the boundary between prostate cancer and normal prostate by the anti-inflammatory effect of vitamin D. Although the relationship between the serum vitamin D levels and multiparametric MRI results needs to be validated in future studies, multiparametric MRI may be recommended for patients with slightly elevated PSA levels and elevated serum vitamin D levels.

There are certain limitations to the current study, including the small number of patients. In addition, this study is a nested case–control study, and the temporal causality is unclear, with a possibility of selection bias. Our center is a municipal hospital operated by the Seoul Metropolitan Government and has a high proportion of patients with low social economic status compared to other hospitals. Even with high PSA level, some patients were unable to perform prostate biopsy and prostate MRI due to difficulties in medical expenses, and it can cause selection bias. The effect of vitamin D level on the incidence of prostate cancer can be studied using prospective cohort studies in the future. Since the vitamin D level in the body changes with exposure to ultraviolet radiation, seasonal variation should be considered. In previous studies, there was a significant seasonal difference in vitamin D level. However, in our study, there was no significant difference in the vitamin D level according to the season (Supplement 1).

## Conclusion

To date, most studies on prostate cancer and vitamin D have been conducted on European Americans and African Americans with a small number of patients. In this study, we found that the incidence of clinically significant prostate cancer might related to low vitamin D level in the Asian population. In the future, a larger population and prospective study are needed.

## Supplementary Information

Supplementary Information.

## Data Availability

The raw data of the study are available after request from the authors.
