# Supplementary material for: The role of the serum 25-OH vitamin D level on detecting prostate cancer in men with elevated prostate-specific antigen levels
Source: Sci Rep. 2022 Aug 18;12:14089. doi: 10.1038/s41598-022-17563-8 (PMC9388499; doi:10.1038/s41598-022-17563-8)
Supplement: Supplementary file 1 — Supplementary Information. [file 41598_2022_17563_MOESM1_ESM.docx]

Supplement 1. Differences in serum vitamin D levels according to seasons

|  | Spring | Summer | Autumn | Winter | *p* value |
| --- | --- | --- | --- | --- | --- |
| 25-OH-VitDlevel (ng/mL) | 18.2$\pm$9.0 | 20.4$\pm$10.2 | 19.5$\pm$7.1 | 19.8$\pm$10.4 | 0.551 |

Supplement 2. MR target lesions according to serum Vitamin D level

|  | Univariate (OR 95% CI) | *p* value |
| --- | --- | --- |
| 25-OH-VitD level | 1.043 (1.016 – 1.070) | 0.002 |
